# Prospective Multicentre Study on the Epidemiology and Current Therapeutic Management of Severe Bronchiolitis in Spain

**DOI:** 10.1155/2017/2565397

**Published:** 2017-03-22

**Authors:** Jose C. Flores-González, Juan Mayordomo-Colunga, Iolanda Jordan, Alicia Miras-Veiga, Cristina Montero-Valladares, Marta Olmedilla-Jodar, Andrés J. Alcaraz-Romero, Miren Eizmendi-Bereciartua, Francisco Fernández-Carrión, Carmen Santiago-Gutierrez, Esther Aleo-Luján, Sonia Pérez-Quesada, Cristina Yun-Castilla, Carmen Martín, Álvaro Navarro-Mingorance, Concha Goñi-Orayen

**Affiliations:** ^1^Unidad de Cuidados Intensivos Pediátricos, Hospital Universitario Puerta del Mar, Cádiz, Spain; ^2^Sección de Cuidados Intensivos Pediátricos, Hospital Universitario Central de Asturias, Oviedo, Spain; ^3^Unidad de Cuidados Intensivos Pediátricos, Hospital Sant Joan de Déu Barcelona, Barcelona, Spain; ^4^Unidad de Cuidados Intensivos Pediátricos, Hospital de Burgos, Burgos, Spain; ^5^Unidad de Cuidados Intensivos Pediátricos, Hospital Universitario Virgen Del Rocío, Sevilla, Spain; ^6^Unidad de Cuidados Intensivos Pediátricos, Hospital 12 de Octubre, Madrid, Spain; ^7^Unidad de Cuidados Intensivos Pediátricos, Hospital Universitario Gregorio Marañón, Madrid, Spain; ^8^Unidad de Cuidados Intensivos Pediátricos, Hospital Universitario Donostia, San Sebastián, Spain; ^9^Unidad de Cuidados Intensivos Pediátricos, Hospital Universitario de Salamanca, Salamanca, Spain; ^10^Unidad de Cuidados Intensivos Pediátricos, Hospital de Jaén, Jaén, Spain; ^11^Unidad de Cuidados Intensivos Pediátricos, Hospital Clínico San Carlos, Madrid, Spain; ^12^Unidad de Cuidados Intensivos Pediátricos, Hospital General Universitario de Alicante, Alicante, Spain; ^13^Unidad de Cuidados Intensivos Pediátricos, Hospital Universitario Regional de Málaga, Málaga, Spain; ^14^Unidad de Cuidados Intensivos Pediátricos, Hospital Virgen de la Salud, Toledo, Spain; ^15^Unidad de Cuidados Intensivos Pediátricos, Hospital Virgen de la Arrixaca, Murcia, Spain; ^16^Unidad de Cuidados Intensivos Pediátricos, Complejo Hospitalario de Pamplona, Pamplona, Spain

## Abstract

*Objective*. To determine the epidemiology and therapeutic management of patients with severe acute bronchiolitis (AB) admitted to paediatric intensive care units (PICUs) in Spain.* Design*. Descriptive, prospective, multicentre study.* Setting*. Sixteen Spanish PICUs.* Patients*. Patients with severe AB who required admission to any of the participating PICUs over 1 year.* Interventions*. Both epidemiological variables and medical treatment received were recorded.* Results*. A total of 262 patients were recruited; 143 were male (54.6%), with median age of 1 month (0–23). Median stay in the PICU was 7 days (1–46). Sixty patients (23%) received no nebuliser treatment, while the rest received a combination of inhalation therapies. One-quarter of patients (24.8%) received corticosteroids and 56.5% antibiotic therapy. High-flow oxygen therapy was used in 14.3% and noninvasive ventilation (NIV) was used in 75.6%. Endotracheal intubation was required in 24.4% of patients. Younger age, antibiotic therapy, and invasive mechanical ventilation (IMV) were risk factors that significantly increased the stay in the PICU.* Conclusions*. Spanish PICUs continue to routinely use nebulised bronchodilator treatment and corticosteroid therapy. Despite NIV being widely used in this condition, intubation was required in one-quarter of cases. Younger age, antibiotic therapy, and IMV were associated with a longer stay in the PICU.

## 1. Introduction

Bronchiolitis is the most frequent cause of lower respiratory tract infection in infants and the predominant reason for admission in children under 1 year of age [1, 2]. Respiratory syncytial virus (RSV) is the most common aetiological agent and the one that determines the seasonal nature of the condition [3]. Around 2%–5% of patients with acute bronchiolitis (AB) require hospital admission; of these, 3%–11% will require admission to a paediatric intensive care unit (PICU) [4]. However, when the group of patients with risk factors for severe bronchiolitis is analysed, this figure reaches 9.4%–50%. Although a series of risk factors for severity have been identified, most patients admitted to the PICU do not present any of these [1, 5].

Despite the significant care demands and high number of hospital admissions, few therapeutic alternatives have been shown to be effective [6]. Numerous studies have shown wide variation in the therapeutic management of moderate AB [7, 8]. Despite a lack of evidence, personal or institutional preferences are often maintained when AB patients require admission to the PICU [7, 8].

High-flow oxygen therapy and noninvasive ventilation (NIV) are increasingly used to the detriment of invasive mechanical ventilation (IMV) [9, 10]. Although studies assessing the use of high-flow therapy in bronchiolitis suggest that it can reduce the work of breathing (WOB) and endotracheal intubation rate, this is not recommended in the latest systematic review [11]. Similarly, the latest systematic review on continuous positive airway pressure (CPAP) concluded that the scant evidence available to support the claim that it reduces the WOB in bronchiolitis is based on poorly designed studies and that no conclusions can be drawn on its effect [12]. There are various indications and modalities of invasive respiratory support in infants with severe AB, but to date no modality has been proven to be superior to any other, although this has not been corroborated in clinical trials.

With respect to the above, the aim of this study was to prospectively determine the epidemiology of patients with severe AB admitted to PICUs in Spain and to describe their therapeutic management.

## 2. Materials and Methods

This is a descriptive, prospective, multicentre study in which 16 Spanish PICUs from the following hospitals participated: Hospital Universitario Puerta del Mar (Cádiz), Hospital Universitario Central de Asturias (Oviedo), Hospital Sant Joan de Déu (Barcelona), Hospital de Burgos, Hospital Universitario Virgen Del Rocío (Sevilla), Hospital 12 de Octubre (Madrid), Hospital Universitario Gregorio Marañón (Madrid), Hospital Universitario Donostia (San Sebastián), Hospital Universitario de Salamanca, Hospital de Jaén, Hospital Clínico San Carlos (Madrid), Hospital Universitario Regional de Málaga, Hospital General Universitario de Alicante, Hospital Virgen de la Salud (Toledo), Complejo Hospitalario de Pamplona, and Hospital Virgen de la Arrixaca (Murcia).

Patients with severe AB who required admission to any of the participating PICUs over 1 epidemiological year (from 1 October 2014 to 15 May 2015) were included; parents or guardians signed an informed consent. Inclusion criteria were (1) infant aged under 24 months with a clinical diagnosis of AB [1, 13], (2) presenting with bronchiolitis classified as severe, and (3) informed consent form signed by the parents or guardians. Exclusion criteria were admission of a patient with AB for reasons of age or episodes of apnoea with no severity criterion, having presented previous episodes of respiratory distress, and refusal of parent or guardian to sign the informed consent form. Bronchiolitis was defined as the first episode of respiratory distress with wheezing and/or crackles associated with symptoms of upper respiratory tract infection during the epidemic period [1]. Criteria for admission to the PICU were defined as follows: severe respiratory failure with a score of >7 on the Wood-Downes-Ferrés scoring system [14] or >10 on the Sant Joan de Déu hospital scale [15] and respiratory acidosis: pH < 7.2, SatO_2_ < 90% with FiO_2_ 40%, cyanosis with oxygen therapy, severe extrapulmonary symptoms, altered level of consciousness, or rapidly progressive disease.

The following variables were recorded during admission: demographic characteristics (medical record number, sex, date of birth, and town/city); stay in PICU with date of admission and discharge and hospital stay after PICU discharge; family history of atopy (atopic dermatitis, asthma, or allergic rhinitis) or smoking; personal history of atopy (atopic dermatitis); type of feeding received so far: breast-fed, bottle-fed, or mixed; presence of risk factors for severity (prematurity, heart disease, bronchopulmonary dysplasia, neuromuscular disease, or immunodeficiency); origin of admission (hospital ward, emergency department, or other hospitals); result of RSV test; reason for admission to PICU (deterioration in clinical condition, presence of risk factors, including age, presence of apnoea, and abnormal blood gas results); analytic result at PICU admission (pH and venous pCO_2_); medical treatment received during the stay in the PICU (adrenaline, salbutamol, corticosteroids, and 3% hypertonic saline solution [3% HSS]); antibiotic therapy and reason for use (clinical deterioration, increased acute phase reactants, radiological image, or others); treatment or prophylaxis with palivizumab; respiratory support received during stay in the PICU (oxygen therapy, high-flow oxygen, NIV, or invasive ventilation); need for high frequency ventilation; need for sedoanalgesia and/or muscle relaxation; complication with hospital-acquired pneumonia or exitus.

The diagnosis of pneumonia was established according to clinical (fever), analytical (increase of acute phase reactants), and radiological (appearance of acute condensation) parameters. For the diagnosis of pneumonia associated with mechanical ventilation, the criteria of the CDC were used.

### 2.1. Statistical Analysis

Variables were collected in a database and analysed using the statistical package SPSS, version 21.

Descriptive results for the quantitative variables were expressed as measures of central tendency and dispersion. The mean and standard deviation were used in the case of variables that followed a normal distribution; otherwise, median and interquartile range were used. Qualitative variables were expressed as number and percentage. Normal distribution of the quantitative variables was assessed using the Kolmogorov-Smirnov test.

Student's *t*-test was used to compare 2 means in the case of parametric quantitative variables with homogeneous variances; if the variables were nonparametric and there was no homogeneity of variances, they were compared using Mann-Whitney *U* test with Bonferroni correction.

For the multivariate analysis, linear regression models were fitted, first manually entering the variables previously found to be statistically significant in the bivariate analysis. Using this model with all the variables, variables that were not statistically significant were deleted one by one using the backward method, removing the nonsignificant variables from the model in each step.

### 2.2. Ethical Aspects

This study was approved by the local ethics committee. The parents or legal representatives of each patient were informed and asked for informed consent to collect the data described.

## 3. Results

**Figure 1:**
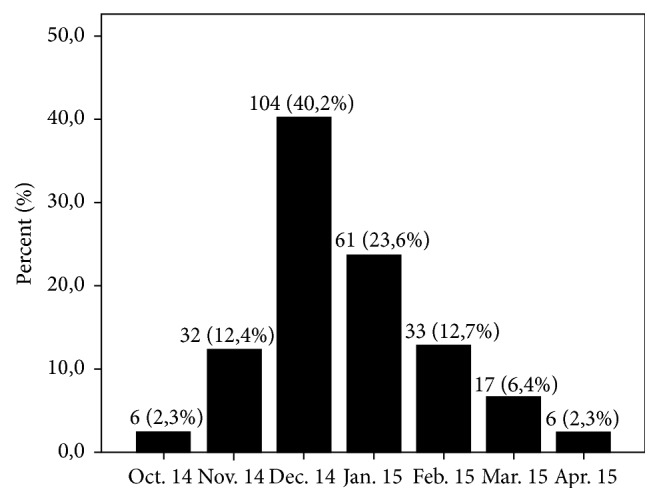
Percentage of admissions for bronchiolitis by month.

A total of 262 patients were recruited in the 16 participating Spanish PICUs; 54.6% were male. Median age was 1 month (0–23). Median stay in the PICU was 7 days, and median hospital stay after admission at PICU was 4 days. Just over one-quarter of patients (26.7%) presented some risk factor for severity, and 14 patients had received palivizumab (only two RSV+). Admission rates were highest in December and January (Figure 1).

The median number of days of respiratory distress until admission to the PICU was 2 days. The main criterion for admission to the PICU was severity (77.2%). Epidemiological and clinical characteristics of the sample are described in Tables 1 and 2. Etiologically, only RSV was detected in the nasal mucus, the causative agent in 78% of the cases. The stay in the PICU of patients who had a family or personal history of interest, compared to those who did not, is shown in Table 3.

Sixty-two patients (23.7%) received no type of nebuliser treatment. The rest received inhaled therapy with adrenaline (38 patients, 14.5%), hypertonic saline with adrenaline (24 patients, 9.2%), salbutamol inhaled or nebulised (23 patients, 8.8%), 3% HSS (11 patients, 4.2%), or several of these during the same stay (104 patients, 39.7%). Almost one-quarter of patients (24.8%, 65 patients) received corticosteroids (mainly methylprednisolone) with a median duration of 4 days (0–13), and 56.5% (148 patients) received antibiotic therapy (Tables 4 and 5). Three patients (1.1%) received treatment with palivizumab during their PICU stay.

**Figure 2:**
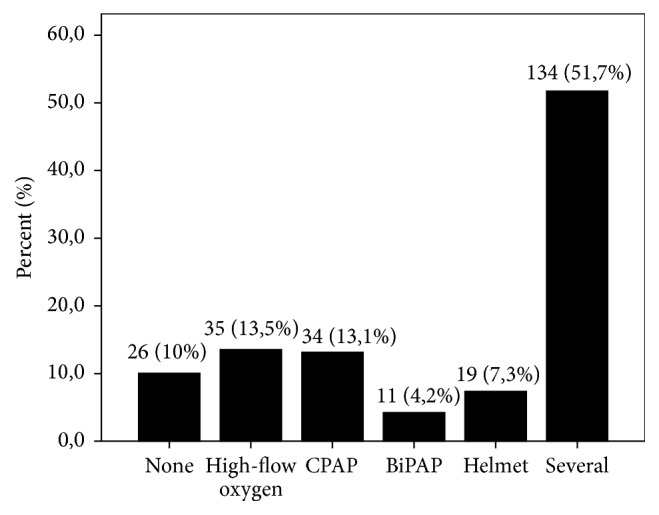
Noninvasive ventilation modalities.

Three-quarters of patients (76%, 199 patients) received oxygen therapy for a median duration of 2 days (0–21 days) and 7 patients (2.7%) received heliox. High-flow oxygen was used in 13.5% (35 patients), and some modality of NIV was used in 75.6% of patients (198 patients) (Figure 2). The modalities selected were as follows: CPAP (53 patients, 20.22%), bilevel positive airway pressure (BiPAP) (11 patients, 4.2%), and several modalities (134 patients, 51.7%).

Sixty-four patients (24.4%) required endotracheal intubation and connection to IMV for a median of 8 days (2–27). Of these, 34 (53.2%) required endotracheal intubation before admission, 10 (15.6%) on the day of admission, and 20 (31.2%) after their first 24 hours of admission. Of the patients who required endotracheal intubation after PICU admission (32), 65.6% received some type of previous noninvasive respiratory support (high-flow oxygen was used in 6.3% (2 patients), CPAP in 15.6% (5 patients), bilevel positive airway pressure (BiPAP) in 18.8% (6 patients), and several modalities in 25% (8 patients)). All intubated patients required sedoanalgesia perfusion for a median of 7 days (2–27); half of these (32 children) also received muscle relaxants for a median of 5 days (2–19). Six patients (2.3%) were diagnosed with hospital-acquired pneumonia. The death rate was 0.4% (1 patient).

The length of stay in the PICU was longer in patients who required IMV (10 days, 4–46) than in those who did not (6 days, 1–21): *p* < 0.0001. Similarly, the stay was shorter in those who required NIV (6 days, 2–21) than in those who did not (10 days, 4–46): *p* < 0.0001.

In the multivariate analysis, 32% of the longer stays were due to younger age, need to start antibiotics, IMV, and muscle relaxation, as these were risk factors that significantly increased length of stay in the PICU (Table 6).

## 4. Discussion

As far as we are aware, this is the first prospective multicentre study designed to describe the epidemiology of severe bronchiolitis cases admitted to PICUs in Spain. In our study, most of the patients admitted with severe AB to the PICU were previously healthy patients with no risk factors and, therefore, were not candidates for receiving prophylaxis with palivizumab [16]. Of those with risk factors, the most common was prematurity, followed by heart disease; the percentage of patients with respiratory disease was lower than that published [17]. Although 26.7% of patients admitted to the PICU had risk factors for severe bronchiolitis, only 5.3% met current criteria for palivizumab vaccination; this figure is higher than that found in other studies, which ranges from 1.9% to 3.7% [18–20]. Presenting some risk factor for severity was not associated with longer stays in the PICU, but there was a significant increase in the length of stay in patients with more siblings. In our study, exclusive breast-feeding did not reduce the stay, as reported by Vereen et al. [21]. The median age on admission to the PICU, percentage isolation of RSV, distribution of incidence of admissions by month, and mean duration of hospitalisation in the PICU were consistent with other epidemiological studies from other countries [16, 17].

This study highlights the wide variation of severe bronchiolitis management, as is described for mild and moderate bronchiolitis in general ward. We found a high frequency of use of some type of bronchodilator, despite current scientific evidence [1]. Furthermore, there was significant variation in the choice of nebulised treatment, from 23% of patients who did not receive any inhaled therapy to 40.8% of patients who received several types of inhaled medication during a single stay. Bronchodilators, salbutamol or adrenaline, have not been shown to improve outcomes, admission rate, or duration of hospitalisation in these patients [22, 23]. In our study, nebulised adrenaline was chosen as the main bronchodilator, likely based on articles that showed a slight short-term improvement in some clinical parameters [24].

Both physiological saline and 3% HSS were used as diluents. The latest systematic review concluded that 3% HSS can reduce the hospital stay in mild and moderate AB [25, 26], although most of the literature published subsequently did not find any benefit in terms of length of stay [26–28]. These studies are not comparable with ours because nonsevere bronchiolitis cases were included.

In recent years, high-flow oxygen and NIV have become the respiratory support of choice in children with moderate-to-severe bronchiolitis, so that most patients with severe AB in Spain are given some type of respiratory support on admission to the PICU, although neither the ideal modality and duration nor best method for weaning from these therapies has been defined. CPAP and high-flow oxygen can improve oxygenation and the WOB and reduce the need for intubation, although this is still questioned due to the contradictory findings of some studies [10, 29]. Evidence to support the use of CPAP or high-flow oxygen is scant and is based on a few poorly designed clinical trials [30]. The latest systematic review did not find sufficient evidence for the use of high-flow oxygen in AB despite being very well tolerated, while its recommendation is based on a single study [31]; the same conclusion was reached for CPAP, similarly based on few clinical trials with a total of 50 patients and contradictory results [32]. In our study, patients with NIV (any modality) had a significantly shorter stay in the PICU.

Almost one-quarter of patients (24.4%) in our study required intubation. These figures are similar to other studies [16, 17, 33]. Intubation is associated with the administration of sedoanalgesia (100% of ventilated patients) and even muscle relaxants (50% of ventilated patients), increasing patients' length of stay. Likewise, as expected, we found that IMV is a risk factor for longer stay in the PICU and that the need for IMV may also actually be a marker of greater severity. Patients who received antibiotics also had a longer stay, probably because they were patients who developed a bacterial overinfection that delayed their clinical recovery.

Our mortality rate was lower than that reported in retrospective studies conducted in France with a mortality rate of 12.8/1000 [17] or in Switzerland with a rate of 11.6/1000 [18] and much lower than the rate of up to 42/1000 reported in series in which populations with risk factors were specifically analysed [34–36]. This may be mainly due to variations in the severity of the different epidemics or the proportion of infants with risk factors for severity admitted to the PICU [37, 38].

This study has several limitations. First, not all patients admitted to PICUs during the epidemic season were included, given the care overload typical of the period, and, second, the voluntary participation in the study could imply some type of selection bias, although the sample is sufficiently large to be representative. The study design and objectives did not allow conclusive relationships to be established as regards the type of noninvasive respiratory support used (HFO, CPAP, and BiPAP) and need for IMV. Although the epidemiology was an objective of our study, most of the PICU participants only tested RSV as an etiological agent, and it is the only variable that we have presented in our study. The reason may be because the study was designed to assess habitual clinical practice and no recommendations or interventions were made. Finally, the hospital-acquired pneumonia rate may not be accurate, given the well-known problems in diagnosing ventilator-associated pneumonia, especially when the patient does not receive invasive ventilation [39].

This study also has some strengths. This is the first prospective Spanish multicentre study to describe the therapeutic management of severe BA. Moreover, it includes a large sample of cases, which means that the information collected accurately represents the current reality.

## 5. Conclusions

In summary, this study highlights the variation currently found in the management of severe AB in Spanish PICUs. We observed very frequent use of medications with no proven therapeutic effect (bronchodilators and, to a lesser extent, corticosteroids) and noninvasive respiratory support, with IMV in almost one-quarter of all children included. Younger age, antibiotic therapy, and need for endotracheal intubation (especially in patients receiving muscle relaxants) were associated with a longer stay in the PICU.

## Figures and Tables

**Table 1:** Epidemiological, family, and personal history characteristics of patients included in the BRUCIP study.

Variable	Value
Sample size (*n*)	262 patients
Sex (male/female)	143 (54.6%)/119 (45.4%)
Age^*∗*^	1 month (0–23 days)
Weight^*∗*^	4.6 kg (1.4–14)
Stay in PICU^*∗*^	7 days (1–46)
Total hospital stay	11 days (3–59 days)
FH of smoking	83 patients (31.7%)
FH of atopy	66 patients (25.2%)
Breast-feeding for at least first 15 days of life	145 patients (55.3%)
Risk factor for severity	70 patients (26.7%)
(i) Premature	(i) 55 patients (21%)
(ii) Heart disease	(ii) 10 patients (3.81%)
(iii) Respiratory disease	(iii) 5 patients (1.90%)
(iv) Neuromuscular disease	(iv) 2 patients (0.8%)
Prophylaxis with palivizumab	14 patients (5.3%)

^*∗*^Median and range.

FH: family history; PICU: paediatric intensive care unit.

**Table 2:** Clinical and analytic characteristics of patients included in the BRUCIP study.

Variable	Value
Origin of patients	(i) Emergency dept.: 30.2% (ii) Hospital ward: 37% (iii) Other hospitals: 31.7%

Onset of respiratory distress until admission to PICU^*∗*^	2 days (0–20 days)

Criteria for admission to PICU	(i) Severity (77.2%) (ii) Blood gases (1.9%) (iii) Apnoea (7.3%) (iv) Presence of risk factors (1.6%) (v) Mixed (6.1%)

pH on admission	7.31 (6.97–7.54)

pCO_2_ on admission (mmHg)	53.35 (23–116)

Positive RSV test	78%

^*∗*^PICU: paediatric intensive care unit; RSV: respiratory syncytial virus.

**Table 3:** Stay in PICU according to presence of personal or family history.

Factors analysed	Stay in PICU in patients with this factor (days)	Stay in PICU in patients without this factor (days)	*p*
Risk factors	8 (2–46)	6 (1–28)	0.203
Prematurity	8 (2–46)	6 (1–28)	0.213
Heart disease	6.5 (3–22)	7 (1–46)	0.9
Atopic patient	6.5 (2–31)	7 (1–46)	0.659
Atopic parents	7 (2–46)	6 (1–28)	0.275
Smoking parents	6 (1–3)	7 (2–46)	0.527
Patient with 1 sibling	7 (2–29)	6 (1–46)	0.208
Patient with 2 or more siblings	8 (3–28)	6 (1–46)	0.014
Vaccinated with palivizumab	8 (4–28)	7 (1–46)	0.592

Statistics test: Mann–Whitney*U* test.

**Table 4:** Nebulised treatment of patients included in the BRUCIP study. PSS: physiological saline solution; HSS: hypertonic saline solution.

Factors analysed	*N*	Duration (days)	Length of PICU stay	*p*
No nebulisation	62 (23.7%)		7.16 ± 5.99	0.141
Salbutamol inhaled	6 (2.3%)	3.58 ± 3.11	11.6 ± 8.35	
Salbutamol nebulised	17 (6.5%)	4.25 ± 6.66	9.18 ± 8.52	
Adrenaline + PSS nebulised	38 (14.5%)	2.06 ± 2.43	8.0 ± 4.35	
Adrenaline + 3% HSS nebulised	24 (9.2%)	4.43 ± 4.27	8.16 ± 3.74	
3% HSS	11 (4.2%)	4.16 ± 3.54	10.0 ± 6.92	
Several nebulisations	104 (39.7%)		7.78 ± 4.18	

**Table 5:** Antibiotic treatment of patients included in the BRUCIP study.

Factors analysed	*N*	Duration (days)	Antibiotic and *N*	Indication
Antibiotic therapy	124	5 (IQR: 4–7)	No antibiotic: 101 (39.1%) Cefotaxime: 84 (32%) Amoxicillin-clavulanic acid: 30 (11.6%) Macrolides: 18 (9.5%) Ampicillin: 13 (5%) Ceftriaxone: 3 (1.2%) Piperacillin-tazobactam: 4 (1.6%) Meropenem: 2 (0.8%) Vancomycin: 5 (2%) Others: 9 (3.5%)	Clinical: 16 (11%) Analytical: 30 (20.5%) Radiological: 14 (9.6%) Mix: 69 (47.3%) Others: 17 (11.6%)

**Table 6:** Data on multivariate analysis.

Variable	*p*	Confidence interval (95%): lower limit	Confidence interval (95%): upper limit
Younger age	0.013	−0.416	−0.049
Antibiotic therapy	0.006	0.456	2.763
Invasive mechanical ventilation	0.004	0.817	4.198
Muscle relaxation	0.000	3.271	7.786
